# The Ethics of Dentistry

**Published:** 1880-08

**Authors:** John H. Coyle

**Affiliations:** Thomasville, Ga.


### ARTICLE V.

### *The Ethics of Dentistry.*

BY JOHN H. COYLE, D. D. S., THOMASVILLE, GA.

(*Read before the Georgia State Dental Society, Session of* 1880.)

"The increase and multiplication of virtues among men resteth chiefly upon societies, well ordained and disciplined."

LORD BACON.

The science of Ethics should constitute as much a part of professional education as any other science essential to his proper equipment for the successful practice of that profession. This is emphatically true, when applied to the profession of dentistry, for there is no profession in which a high toned moral code is more imperative.

There is certainly no profession which offers more temptations to swerve from the path of strict integrity; so many questions of duty to others daily arising, while self-interest is at war with honest conviction. Hence it happens that the young men entering the profession have the need of that moral courage, which is only attained by those of riper years and experience. If we will but keep in mind the golden rule we can never go very far out of the right path. To do this, however, requires a firm, unflinching adherence under all circumstances to *high moral principle.* This will illumine the darkest path we may be called upon to tread, and chase away the darkest clouds of uncertainty. This principle has been likened unto the spear of the Guardian Angel of Paradise:

"No falsehood can endure
Touch of celestial temper, but turns,
Of force to its own likeness."

It would be easy to declaim at great length, in a general way, and erect an ideal standard of Ethics, and leave each individual to make the application in special cases. It is a difficult thing, however, for the majority of men, in practice, to determine the precise extent to which principle is involved, and just how far duty and interest come in conflict.

These questions have to be met and decided, by none more frequently than the members of our profession. The responsibility, professional and moral, of the practitioners of dentistry, grow out of their duties, first to the people, second to the medical profession, and third to each other, and it is in this order that I propose to treat the subject.

We should never lose sight of the fact that it is *necessity* that places a single patient in our care. No person would undergo the tedious and oftentimes painful operations of the dentist, unless it was an *unavoidable necessity.* We should therefore use all means at our command to make our operations as bearable as possible. The public have a right to demand that we shall furnish a comfortable chair and the best instruments that can be obtained, together with the purest and best materials known and attainable for them. They pay for our services, and have a right to our very best skill and most faithful performance of what we undertake for them. As far as their knowledge of dentistry goes, they are like unto children, and are altogether in our hands. They have to rely upon our honesty and upon our judgment, and when they seek our advice the truth and nothing but the truth should be given under all circumstances. It is most frequently the case that the patient is more than willing to be "gulled," and it is only a question of moral firmness with the dentist. If he is not governed by high moral principle, he will undertake operations at the urgent request of the parties themselves, which he *must* know cannot result in any benefit, except the pecuniary one to himself. This may be a temporary advantage, but sooner or later will injure the dentist who pursues such a course, as well as bring reproach on an honorable profession. We should cultivate an amiable condescension toward all. It is not to be presumed that our patients are very far advanced in the special pathology of the teeth, as is abundantly shown by their questions, and we should patiently endeavor to give them a plain reason in plain English language for our course. How often do people apply for the filling of a tooth, which is found, on examination, to be devitalized with a discharge from it, the fœtor of which is so disgusting and almost unbearable, and when we tell them that it will not do to fill that tooth in that condition, what a stare of incredulity on their faces! And when we explain to them the condition of that tooth, and show them that it is absolutely essential to give it a preliminary treatment before it can be filled, how surprised they are, saying they thought that "a tooth never ached after the nerve was dead." How easy it is to take advantage of such a patient! But the man who would do it, who claims to be a dentist, you all know is either a *fool or a knave.* The public have a right, also, to expect that a dentist shall have an ordinary English education, and a fair acquaintance with the current literature of the day. In this country, I mean the Southland, only the better class of people apply for dental operations. They are, as a rule, people of cultivation and refinement, and how acceptable it must be to them to find that their dentist is a man after their own order, not only a skillful Dr. Forceps, but an agreeable and accomplished gentleman besides. John Ruskin says that "there is rough work to be done, and rough men must do it; there is gentle work to be done, and *gentlemen* must do it." I believe that this applies with emphatic force to dentists.

Our duties to the medical profession are of a less complicated character; though if one attached a great deal of importance to what has been said in this connection during the last few years, he would feel otherwise. To my mind there can be no possible clash of interests, except an apparent pecuniary one, and that does not merit a serious consideration. The dentist is confined both by his attainments and his license to certain circumscribed bounds of practice, and he is not sound in Ethics who desires to overstep these limits without the necessary qualifications and license of law so to do. This limitation of practice is embraced in any and all operations on the teeth and associate parts. In order that the general practitioner and the public may yield to him this practice to the exclusion of the M. D., he must demonstrate his qualifications to more fully meet the requirements of this practice. There will then be no longer any competition for it on the part of the medical men, from the simple fact that the public will voluntarily give it to the skillful specialists—the qualified dentist. We should remember that the medical practitioner is entitled to our profound respect and earnest co-operation. He has to deal with the hydra-headed monster, disease, in all its manifestations, as it crops out and shows itself throughout the entire system. Our knowledge of the special pathology of the mouth alone, should teach us the Herculean task he has before him. On the other hand, we have a right to demand of the medical practitioner a recognition of the superior attainments and advantages of the qualified dentist, for the treatment of dental lesions, and under this view it is a selfish spirit alone that would tempt them to interfere in our special practice. I come now to the last division of the subject: the duty of dentists to their professional brethren. And just here in the beginning let me say that there is a greater disregard of all principles of Ethics here than in either of the preceding sections. It is this brutal ignoring the commonest principles of decency and morals by a large number of dentists, so-called, that has often caused our cheeks to burn with a blush of shame, that we too, being dentists, would be classed with such men. Until a very recent date, the greatest accomplishment of the majority of those engaged in the practice of dentistry in this State, was a *full capacity for running down* other dentists. And there are dentists in Georgia in this day who tell their patients that "they are the only dentists in the Southland who could do that job that way." Dentists of this turn of mind doubt even the possibility of Shakespeare's lines, that

> "There are more things in heaven and earth, Horatio,
> Than are *dreamt* of in your philosophy."

What a calamity to ourselves, as well as those who come into our hands, to get into a "rut" of this kind. We should look around us and recognize the fact that this is an age of *unceasing* activity. The printing press, with its teeming millions of adjuncts, is spreading the truth and the light to millions besides our "one self," and just as the sun, in all his glorious effulgence, is to be seen by other eyes, so are the sources of professional information and professional attainments open to all. The greatest evidence that can be given of a total want of the apprehension of the first principles of dental ethics is this expression of absolute professional egotism, big *I* and *little* you. The time was when the pioneers of Dental education came forth to battle with the hosts of ignorant quacks and self-dubbed doctors, that a moderate boast of superior attainments might have been allowable; but, gentlemen, that time is past, and my heart's desire and prayer to God is that it may never return, but rather that the ethics of the profession may become deeply implanted in the hearts and minds of every dentist in this land.

Gentlemen, the opening words of this paper declare that the "increase and multiplication of virtues among men resteth chiefly upon societies well ordained and disciplined." I believe this to be a profound *truth*, and in view of the great moral responsibility resting on this body for the advancement of virtues among its members, it behooves you to *look well to your discipline.* This is the rock of your strength in your efforts for the future. Your published code of ethics is all that can be desired, provided it is sustained by a rigid enforcement of its provisions on your individual members. A strict enforcement of this code, without fear or favor, will result in honor to yourselves, the advancement of our profession, and confer a lasting benefit on the people of this State. In the very first Article of our Constitution, Section 2, we declare that one of the purposes we have in view is the elevation of the character of dentists. We have only to look at the results of our past efforts to encourage us for the future conflict. In the brief space of eleven years we have attracted to our body the majority of the respectable dentists of the State, who have signified their determination to yield obedience to our system of Ethics. Through our influence Dentistry, which hitherto had no legal status, has been brought into intimate relationship with law and ethics. The people have begun to recognize the truth as it is—that your work is as much for their interests as it is for our own. Rise, then, to the high plane of your duty; insist upon a strict interpretation and a strict enforcement of our code of Ethics, and I promise you that you will meet with a hearty response from the people. Of course there are hundreds of "Cheap Johns," "Bush Whackers," "Quacks and Pretenders," all embraced under the head of Ruskin's "fee first men," who will cry you down, and represent you as a "red tape body," but fear not. The truth is mighty, and *must* prevail, for

"Truth, crushed to earth, shall rise again,
The eternal years of God are hers;
But error, wounded, writhes with pain
And dies among her worshippers."

—*Dental Luminary.*

